# The More the Better?—Vitamin E TPGS as a Release Enhancer for Ritonavir/PVPVA Amorphous Solid Dispersions

**DOI:** 10.1021/acs.molpharmaceut.5c00620

**Published:** 2025-07-27

**Authors:** Ineke Fahrig, Stefanie Walter, Samuel Kyeremateng, Matthias Degenhardt, Gabriele Sadowski, Christoph Brandenbusch

**Affiliations:** † Department of Biochemical and Chemical Engineering, Laboratory of Thermodynamics, TU Dortmund University, Emil-Figge-Street 70, Dortmund D-44227, Germany; ‡ AbbVie Deutschland GmbH & Co. KG, Development Sciences, R&D, Knollstraße, Ludwigshafen am Rhein D-67061, Germany

**Keywords:** amorphous solid dispersion, vitamin E TPGS, release, phase separation

## Abstract

Amorphous solid dispersions (ASDs) are state-of-the-art formulation strategies for improving the solubility and release of poorly water-soluble, small-molecule active pharmaceutical ingredients (APIs). However, high drug loads (DLs) in ASDs can lead to phase-separation phenomena, resulting in eventually incomplete/collapsed API release, classically referred to as loss of release. This study investigates the role of the surfactant d-α-tocopheryl polyethylene glycol succinate (Vitamin E TPGS) in mitigating this phenomenon in ASDs composed of ritonavir and poly­(vinylpyrrolidone-co-vinyl acetate) (PVPVA). As part of the investigations, we developed an improved sampling protocol to differentiate between ASD components molecularly dissolved and those released as nano droplets into the aqueous medium. The results showed that the addition of 3 wt % Vitamin E TPGS enhances release ability up to 30 wt % DL, compared to 25 wt % for the surfactant-free ASD. This enhancement is attributed to Vitamin E TPGS’s ability to stabilize discrete RIT-rich domains in the ASD during phase separation. However, at 40 wt % DL, even high Vitamin E TPGS concentrations (up to 10 wt %) did not lead to full release of the API. This indicates that the aforementioned stabilization mechanism failed and could be traced back to a change in the phase separation behavior above an upper limit of Vitamin E TPGS concentrations. This study thus provides insights into the complex release mechanisms of high-DL ritonavir ASDs and the critical role of surfactants such as Vitamin E TPGS.

## Graphical abstract

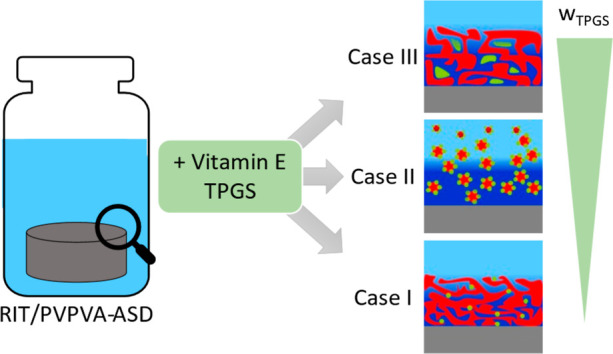

## 1 Introduction

Amorphous solid dispersions (ASDs) are one of the premier pharmaceutical formulation strategies for small-molecule active pharmaceutical ingredients (APIs) with low aqueous solubility.^1^ It is well-known that the amorphous API has a higher apparent aqueous solubility compared to the crystalline form but tends to recrystallize due to its thermodynamic instability. To preserve the advantages of the amorphous state in terms of solubility, the amorphous API is dissolved in an amorphous polymer matrix, forming an ASD. If selected correctly, the polymer delays/hinders recrystallization of the amorphous API.^2–4^ In addition, using an ASD as a formulation technique often enhances the release behavior compared to that of the crystalline or pure amorphous API (if at all physically stable). For example, ASDs with the polymer poly­(vinylpyrrolidone-co-vinyl acetate) (PVPVA) showed an increased release of the APIs ritonavir (RIT)^5,6^ and felodipine^7^ compared to the pure amorphous API. From a formulation perspective, two important aspects need to be considered: (1) In terms of bioavailability, the API should release completely (Figure 1 A). (2) Formulations with high drug load (DL) are desirable, as this decreases tablet size and/or the number of tablets to achieve the required dose of the API.

**Figure 1:**
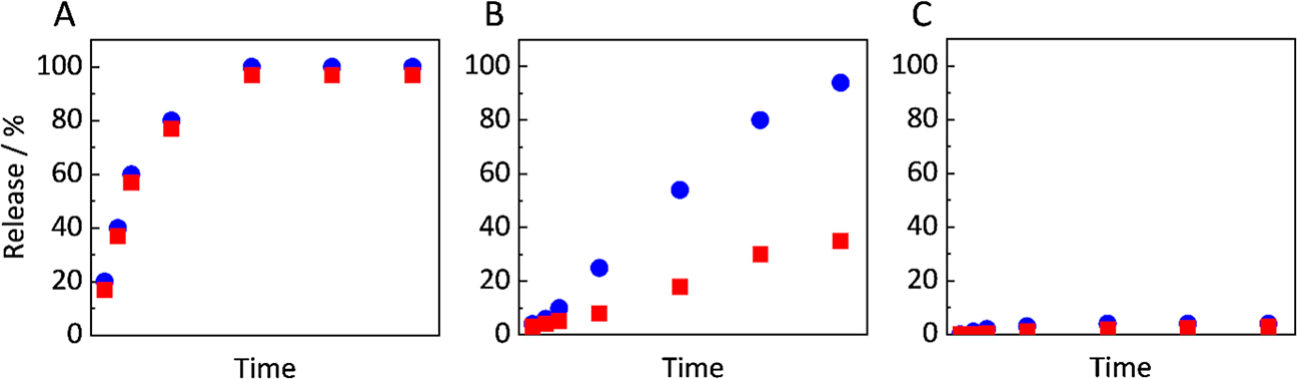
Schematic release profiles of an API (red squares) and a polymer (blue circles) with complete API release (A), incomplete API release (B), and minimal API release (C).

Unfortunately, several studies have shown that the applicable DL has an upper limit that is specific to a particular system.^5–11^ Exceeding this upper DL limit leads to a loss of release (LoR) of the API (see Figure 1B,C), meaning API release drops significantly (with only minimal release remaining) or even collapses completely. As a subset of the LoR, Taylor et al. introduced the term Limit of Congruency to describe the DL at which API and Polymer no longer release congruently (Figure 1 B).^5,12^ For example, ASDs containing RIT and PVPVA still showed complete release of the API in phosphate buffer (pH = 6.8, 37 °C) at a DL of 25 wt %, while at 30 wt %, DL incomplete release behavior (Figure 1B) was observed.^5^ At even higher DLs (up to 40 wt %), the API release collapses significantly, resulting in minimal release of both the polymer and the API (see schematically in Figure 1C).^5^

Previous studies by other researchers and by our group showed that LoR can be primarily attributed to two different mechanisms: (1) Rapid crystallization of the supersaturated API from the amorphous state or (2) liquid–liquid phase separation (LLPS) at the ASD–water interface.^6,10,11^ The ASD phase behavior upon contact with the aqueous bulk phase, especially the LLPS, can be visualized/illustrated from a thermodynamic perspective using a ternary phase diagram,^6^ as shown in Figures 2 and 3. Two main release mechanisms can be differentiated depending on the initial DL of a dry ASD.

**Figure 2:**
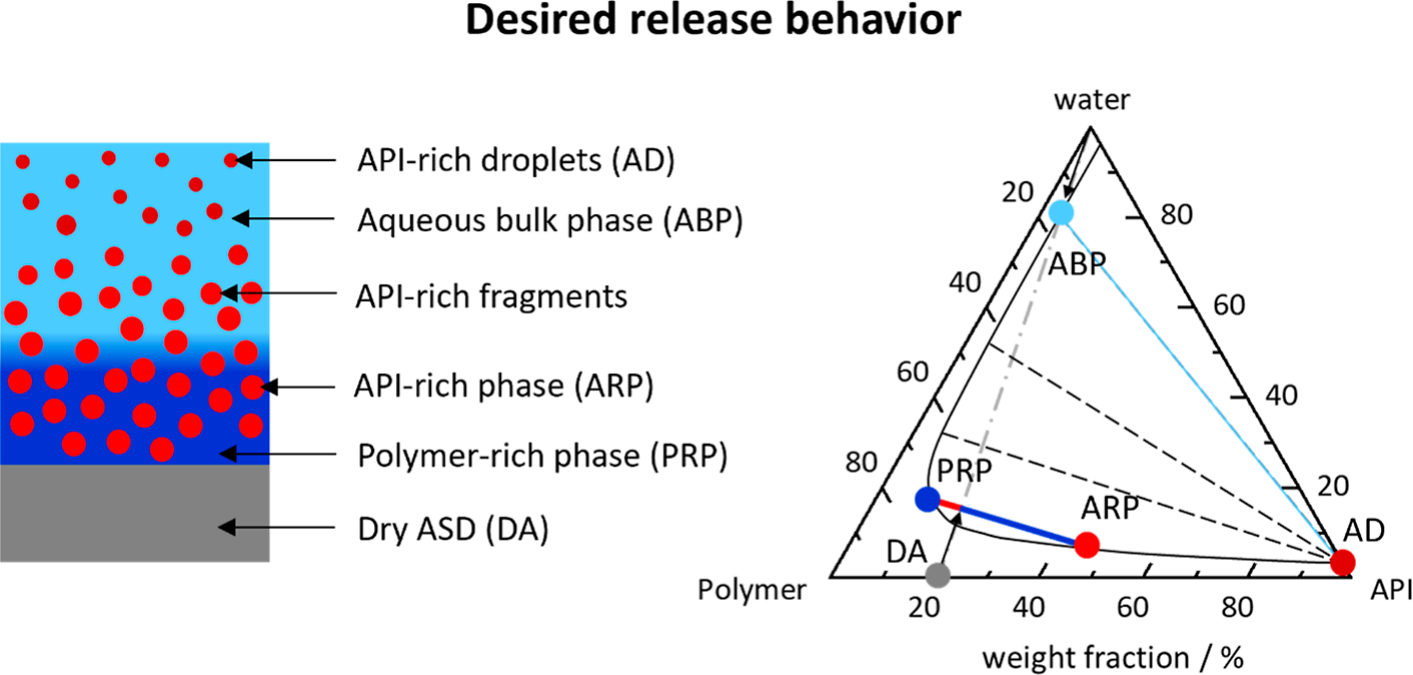
Schematic illustration of the water-induced phase separation in the ASD and schematic ternary phase diagram of API/polymer/water for the desired release behavior. In the phase diagram, the black solid line indicates the miscibility gap, the black dashed lines are the corresponding tie lines, and the gray dash-dotted line (with arrows) represents the hydration pathway. The gray symbol indicates the dry ASD (DA), the dark blue symbol the polymer-rich phase (PRP), and the light red symbol the API-rich phase/released fragments (ARP). The light blue symbol indicates the aqueous bulk phase (ABP), and the dark red symbol indicates the API-rich droplets (AD).

**Figure 3:**
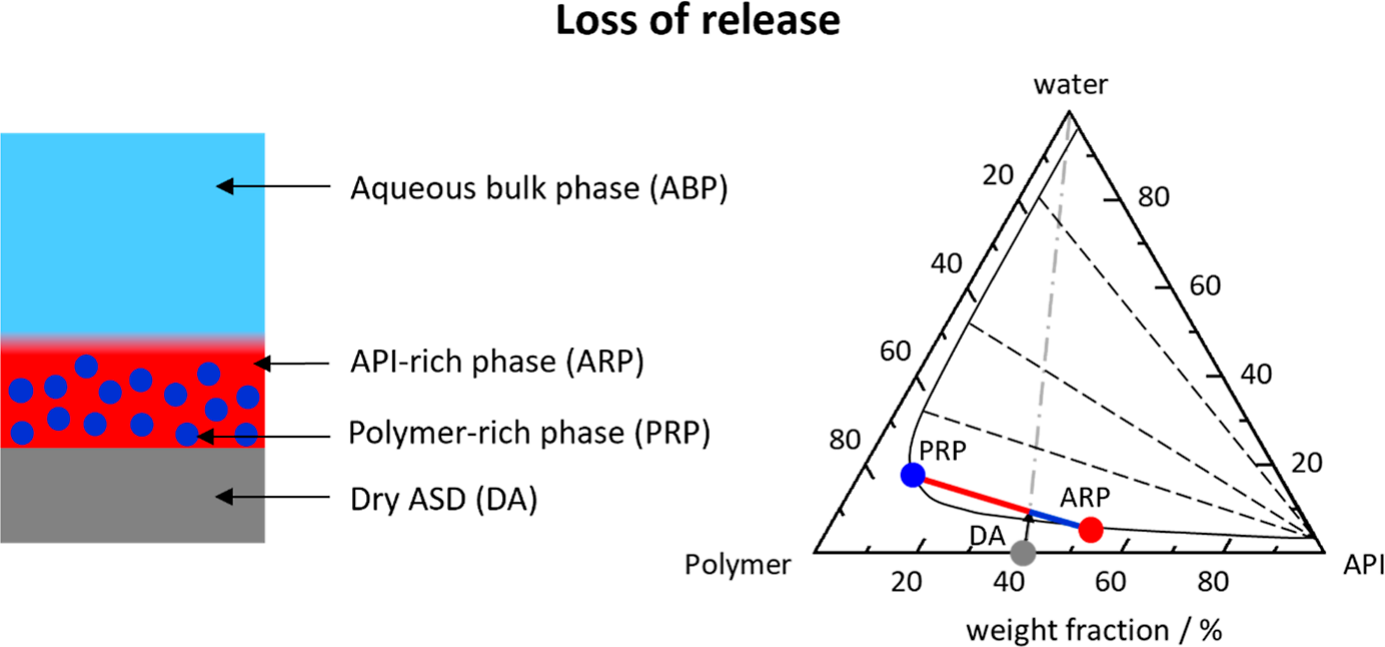
Schematic illustration of the water-induced phase separation in the ASD and schematic ternary phase diagram of API/polymer/water above the LoR. In the phase diagram, the black solid line indicates the miscibility gap, the black dashed lines are the corresponding tie lines, and the gray dash-dotted line (with an arrow) represents the hydration pathway. The gray symbol indicates the dry ASD (DA), the dark blue symbol the PRP, and the light red symbol the API-rich phase/released fragments (ARP).

### 1.1 Desired Release Behavior

Upon contact with the aqueous bulk phase, a dry ASD with a given (low) DL (e.g., 20 wt %, [DA] Figure 2) absorbs water. Once enough water is absorbed and the miscibility gap is reached, LLPS occurs, resulting in a hydrophobic API-rich phase ([ARP] Figure 2) and a hydrophilic polymer-rich phase ([PRP] Figure 2) in the ASD. The mass ratio of the API-rich phase to polymer-rich phase can be determined from the corresponding tie line in the phase diagram using the law of averted levers and is crucial with respect to explaining the LoR phenomenon.

For small DLs, the polymer-rich phase is the continuous phase (the blue lever is longer than the red lever in Figure 2). Detailed microscopic investigations by the Taylor group confirmed dispersed API-rich phases in a continuous polymer-rich phase (shown schematically in Figure 2, left).^8,13^ The polymer-rich phase, having a high aqueous solubility, dissolves quickly into the aqueous bulk phase. The dispersed API-rich phase (having a low aqueous solubility) is only partially dissolved but is mostly released as API-rich “fragments” into the aqueous bulk phase. These released API-rich fragments strive for thermodynamic equilibrium with the aqueous bulk phase. They continuously dissolve until the amorphous solubility of the API in the aqueous bulk phase is reached ([ABP] in Figure 2). Once the amorphous solubility is exceeded, LLPS occurs, resulting in API-rich droplets ([AD] in Figure 2) that are in thermodynamic equilibrium with the aqueous bulk phase ([ABP] in Figure 2). The composition of the API-rich fragments consequently changes during the course of their partial dissolution into the state of API-rich droplets.

### 1.2 LoR

At higher DLs, the amount of the hydrophobic API-rich phase exceeds the amount of the polymer-rich phase formed during LLPS in the ASD^13^ (the red lever is longer than the blue lever in Figure 3). The ASD thus separates into an API-polymer structure ([ARP] Figure 3) continuous API-rich phase ([ARP] in Figure 3) in which the polymer is now dispersed.^8,14,15^ As in the previous case, the polymer at the interface dissolves due to its high aqueous solubility, leaving the remaining (continuous) API-rich phase to form a porous network (compared to the previously dispersed fragments; see Figure 2). The API dissolution is slow due to the low aqueous solubility, leading to the formation of an API-rich layer at the ASD-water interface.^15^ This hinders further dissolution of the polymer, and LoR is observed.^5,8,10^

To circumvent LoR at high DLs, the addition of surfactants has been shown to be a promising approach.^7,16–20^ For example, adding 4.7 wt % d-α-tocopheryl polyethylene glycol succinate (Vitamin E TPGS) to ASDs with RIT and PVPVA increases the LoR from 25 wt % (without Vitamin E TPGS) to 29 wt % DL.^17^ Comparable results have been obtained by Saboo et al.^20^ on the influence of Vitamin E TPGS on felodipine-PVPVA ASDs. Adding 5.5 wt % Vitamin E TPGS to the felodipine-PVPVA ASD increases the LoR from 15 wt % DL to 45 wt % DL.

Several studies indicate that Vitamin E TPGS enhances API release by preventing the formation of the continuous API-rich phase during LLPS in the ASD.^7,16–20^ It is suggested that surfactants adsorb at the API–polymer interface during LLPS, stabilizing these domains and thus inhibiting their coalescence.^18,19^ Other researchers indicated that surfactants like SDS may also change the miscibility of the API in the API-polymer–water system, thereby inhibiting LLPS completely.^19^ Another possibility is that Vitamin E TPGS modifies the location of the critical point of the two-phase region, consequently shifting the phase inversion threshold to higher drug loads. However, the exact release mechanism is not yet well understood or experimentally proven.

In this work, we focused on Vitamin E TPGS as a potential release enhancer in RIT/PVPVA ASDs. We investigated whether increasing concentrations of Vitamin E TPGS lead to enhanced API release in ASDs with high DL. To achieve this, we conducted release experiments on ASD films in water at 37 °C with varying DL and polymer/surfactant ratios. Our results demonstrate that beyond a certain concentration threshold, increasing the amount of Vitamin E TPGS did not increase the release of RIT from ASDs with high DLs. Additionally, we developed an improved sampling protocol to gain further insights into the Vitamin E TPGS-assisted release of ASDs consisting of RIT and PVPVA. Previous studies (also from our group) focused on the phase compositions during LLPS in the ASD^8,10,21^ ([PRB] and [ARP] in Figures 2 and 3), as well as on the (global) release behavior. In these works, the compositions of unfiltered^5,7,9,12^ or 1 μm filtered samples^22^ of the aqueous bulk phase were investigated. In contrast to this, we now aim to distinguish between the amounts of ASD components released into the aqueous bulk phase as nano droplets and the ones molecularly dissolved. Furthermore, we take into account that the API-rich fragments ([ARP] in Figure 2) will change their composition upon release into the aqueous bulk phase as they strive for API-rich droplets ([AD] in Figure 2), which are in contact with the aqueous bulk phase.

## 2 Materials and Methods

### 2.1 Materials

RIT was supplied by AbbVie Deutschland GmbH & Co. KG (Ludwigshafen, Germany). PVPVA was obtained as Kollidon VA 64 from BASF SE (Ludwigshafen, Germany). The surfactant Vitamin E TPGS was purchased from Merck KGaA (Darmstadt, Germany). Acetonitrile was purchased from VWR International S.A.S (Rosny-sous-Bois, France) and methanol from Merck KGaA (Darmstadt, Germany). Water was purified with the Milli-Q Advantage A10 purification system from Merck KGaA (Darmstadt, Germany). To ensure comparability between experiments and modeling, we relied on Millipore water instead of aqueous buffer solutions. All chemicals were used without further purification.

### 2.2 ASD Sample Preparation

ASDs composed of RIT, PVPVA, and Vitamin E TPGS were produced by vacuum compression molding (VCM). The DLs of the ASDs were set to 20, 30, and 40 wt %. The amount of PVPVA was adjusted based on the amount of Vitamin E TPGS, which was set to 0, 3, 7, and 10 wt %. Eight different ASD compositions were investigated. All measurements were performed in triplicate. Initial physical mixtures of the components were homogenized by ball milling. A total amount of 1 g was placed into the stainless-steel cup of a Pulverisette 23 ball mill from Fritsch GmbH (Idar-Oberstein, Germany). The entire cup (including a steel ball) was shaken three times for 3 min at 50 Hz. Milling was interrupted for 1 min between each run to dissipate the heat.

We produced round films with a thickness of 1 mm by filling 0.06 g of the milled mixture into an Ø8 mm VCM tool from MeltPrep GmbH (Graz, Austria). A low-pressure lid was placed on the VCM tool to prevent material loss in low-viscosity mixtures. The mixture was melted for 15 min at 150 °C under vacuum, followed by cooling to room temperature for 15 min using the cooling unit of the MeltPrep device. The films were confirmed to be amorphous after preparation, indicated by a single glass transition temperature in modulated differential scanning calorimetry measurements and the absence of a melting peak. An exemplary result for an ASD with 40 wt % DL and 10 wt % Vitamin E TPGS is shown in the Supporting Information (see Figure SI6). Before use, all films were stored under a vacuum at 25 °C to avoid water sorption.

### 2.3 Release Experiments

The release experiments were performed in a dissolution apparatus consisting of an 8 mm holder for the ASD film and a magnetic stirrer, which is set to 100 rpm. The holder ensures a constant tablet surface exposed to the dissolution medium. The release experiments were conducted in 100 mL Millipore water at 37 °C to eliminate the influence of additional substances (as phosphate buffer) on release mechanisms. The water temperature was adjusted by the Ecoline RE 304 thermostat from Lauda GmbH & Co KG. (Lauda-Königshofen, Germany). Seven samples (5 mL each) were withdrawn periodically from the medium and replaced with fresh water with equal volume over a total of 120 min. The samples were analyzed via HPLC and dynamic light scattering (DLS).

### 2.4 Concentration Determination via HPLC

All samples were quantified by HPLC using an Agilent 1200 HPLC instrument from Agilent (CA, USA). The concentration of RIT was measured using an EC-C18 Poroshell 120 column (2.1 cm × 100 mm, 2.7 μm pore size) from Agilent. The mobile phase consisting of water/acetonitrile (60/40 v/v %) was pumped at a flow rate of 0.2 mL min^–1^. The injection volume was 10 μL. RIT was detected using an ultraviolet (UV) detector at a wavelength of 240 nm. The calibration curve was established between the concentration ranges 0.003 and 0.15 g L^–1^ with an R^2^ value of 0.994.

The Vitamin E TPGS concentration was also quantified using an EC-C18 Poroshell 120 column (2.1 mm × 100 mm, 2.7 μm pore size). The mobile phase consisting of acetonitrile/methanol (80/20 v/v %) was pumped at a flow rate of 0.4 mL/min. The injection volume was 30 μL. Vitamin E TPGS was detected using a UV detector at a wavelength of 210 nm. The calibration curve was obtained for the concentration range between 0.001 and 0.07 g L^–1^ with an R^2^ value of 0.993.

The concentration of PVPVA was determined using a 300 mm × 8 mm A2500 Viscothek SEC column from Malvern Panalytical (Malvern, UK). The mobile phase consisting of water/methanol (70/30 v/v %) was pumped at a flow rate of 0.4 mL/min. The injection volume was 50 μL. PVPVA was detected with a UV detector at a wavelength of 205 nm. The calibration curve was obtained for the concentration range between 0.05 and 0.47 g L^–1^ with an R^2^ value of 0.999.

### 2.5 Determination of Hydrodynamic Radius and Particle Size Distribution

We determined the hydrodynamic radius (*R*_h_) and the size distribution of the droplets in the samples at each time point via DLS. For this purpose, 100 μL of each sample was loaded into a 384-well plate and analyzed using the DynaPro Plate Reader III of Wyatt Technology Corporation (CA, USA). For each measurement, 10 acquisitions were recorded at 25 °C, each lasting 2 s. Considering the number size distribution, the collected data were evaluated using the Dynamics software (Wyatt Technology Corporation, Santa Barbara, CA).

### 2.6 Determination of Sample Crystallinity by PXRD

To verify the amorphous or crystalline state after the release experiments, ASDs that were not completely released were examined by powder X-ray diffraction (PXRD). The experiments were run in the MiniFlex 600 from Rigaku (Tokyo, Japan) with Cu K_α_ irradiation at 40 kV and 14 mA. We measured at 5° min^–1^ in the range of 5°<2θ < 30° and a step size of 0.02°.

## 3 Results

### 3.1 Improved Sampling Protocol to Characterize the Released RIT-Rich Fragments and Droplets

To distinguish between the molecularly dissolved amounts of ASD components, released RIT-rich fragments, and RIT-rich droplets in the dissolution medium, we developed an improved sampling preparation/fractionation protocol prior to sample analysis. The samples taken during the release experiments (see Section 2.3) are separated into two aliquots. The first aliquot (1 mL) is diluted 1:1 v/v % with methanol to avoid recrystallization of RIT and to ensure the dissolution of all released RIT-rich fragments and droplets. The concentration determined afterward using the HPLC method described in Section 2.4 represents the total (released) amount of all components in the aqueous bulk phase.

The second aliquot (2 mL) is filtered using a 0.1 μm PVDF syringe filter with low protein binding (Berrytec GmbH, Harthausen, Germany) prior to HPLC analysis (see Section 2.4). Additional details on the validation of the sampling protocol are provided in the Supporting Information. Compared to other studies,^22,23^ we chose a (smaller) pore size of 0.1 μm to remove all particles (that are the RIT-rich fragments and droplets) from the aqueous bulk phase. DLS data showed an *R*_h_ < 10 nm in filtered samples, ensuring that all particles were removed (data not shown here). The filtered solutions were analyzed via HPLC. The concentrations determined represent the amounts of ASD components molecularly dissolved in the aqueous bulk phase. Examining the released as well as the dissolved amounts can reveal two profile cases depending on the solubility of the components in the aqueous bulk phase (see Figure 4).

**Figure 4:**
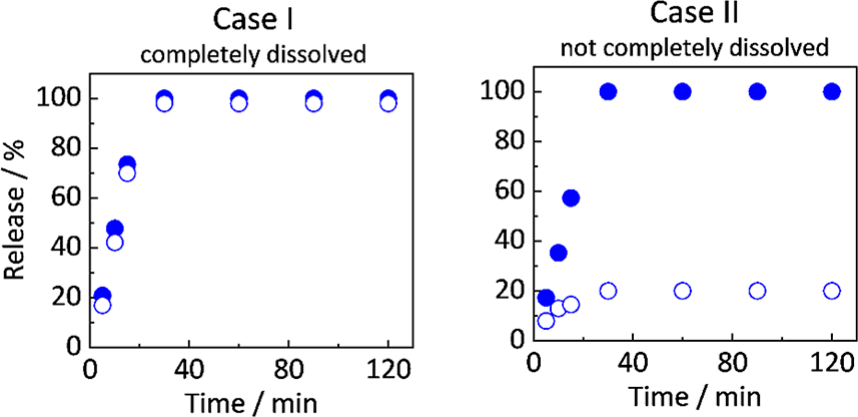
Example release profiles. Filled circles indicate the overall amount of a released component, and empty circles indicate the molecularly dissolved amount of the same component. Case 1: Released amount equals the molecularly dissolved amount. Thus, the component is completely molecularly dissolved in the aqueous bulk phase. Case II: Released amount exceeds the molecularly dissolved amount. In the absence of API crystals, the dissolved amount plateaus at the amorphous solubility of the API. The difference between released and dissolved API indicates the amount of API present in the aqueous bulk phase as amorphous LLPS droplets.

In Case I, the molecularly dissolved amount of a component increases over time until a plateau is reached. This indicates that this component has reached its amorphous solubility in the aqueous bulk phase. The total released amount of the same component soon exceeds the amount of the molecularly dissolved component, suggesting the presence of either fragments or droplets in the medium. In comparison, for Case II, the dissolved amount of a component equals its total released amount. This suggests that the component is completely dissolved and is not enriched in fragments or droplets.

### 3.2 Release of Surfactant-Free ASDs

To investigate the influence of DL on the presence of RIT-rich fragments or RIT-rich droplets in the aqueous bulk phase, we investigated the release of RIT/PVPVA ASDs with three different DLs (namely, 20, 30, and 40 wt %) using our improved sampling protocol. The analysis of the amounts of RIT and PVPVA (after fractionation) was carried out as described in Section 2.3. For validation purposes, we also examined the composition of the particles removed with the 0.1 μm filter. For this purpose, the filter was submerged in 1 mL of methanol for 24 h. The concentration determined via HPLC represents the average composition of the released RIT-rich fragments and RIT-rich droplets.

The hydrodynamic radii (*R*_h_) were measured using DLS (see Section 2.5) to determine the (change) in droplet size/mean droplet diameter in the aqueous bulk phase over time. The results on *R*_h_ are illustrated in Figure SI1 (Supporting Information). Figure 5 illustrates the results for the release experiments (RIT/PVPVA) at 20, 30, and 40 wt % DL. For better visualization, only the data points for the total released amount of PVPVA are shown, as released and dissolved amounts were identical. The detailed graphical illustration, including differentiation between dissolved and release amounts, is shown in Figure SI2 (Supporting Information).

**Figure 5:**
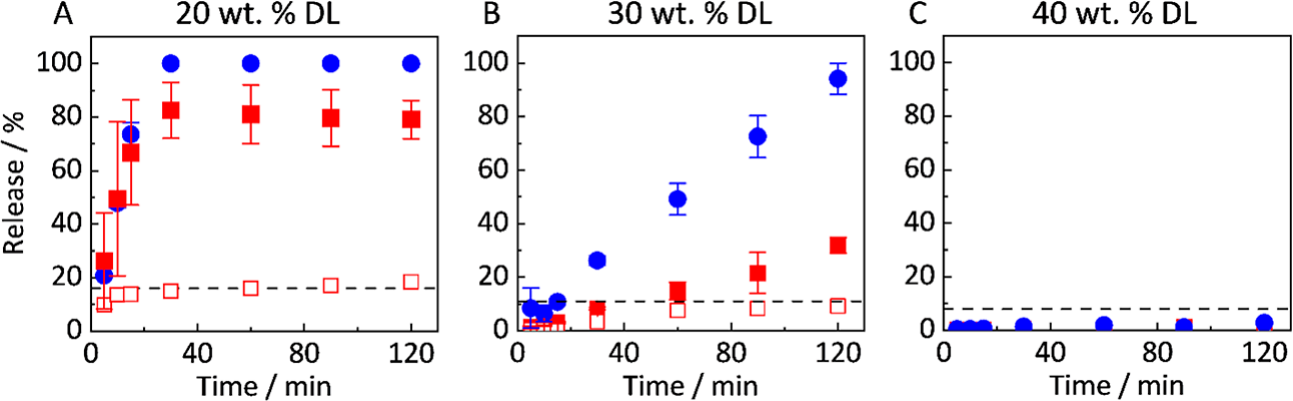
Release profiles of RIT/PVPVA ASDs with 20 wt % DL (A), 30 wt % DL (B), and 40 wt % DL (C). Filled symbols indicate the released amount, and empty symbols indicate the molecularly dissolved amount of RIT (red squares) and PVPVA (blue circles). The dashed line represents the amorphous solubility of RIT measured in this work.

The results (Figure 5 A) reveal that at 20 wt % DL, PVPVA is completely dissolved (open and solid symbols overlay, see Figure SI2 in the Supporting Information). In contrast, RIT is only molecularly dissolved up to its amorphous solubility (Figure 5A and Table 1). The remaining amount of RIT is present in released fragments/droplets, which was also indicated by the turbidity of the samples taken from the aqueous bulk phase. For all time points, the particles (fragments/droplets removed from the filter and analyzed by HPLC after dissolution in methanol) contained almost pure RIT, with the concentration of PVPVA being below the detection limit of the HPLC (results not shown). It is thus obvious that the fragments released from the ASD almost instantaneously changed their composition to that of the droplets (in equilibrium with the aqueous bulk phase). DLS data show the appearance of particles (*R*_h_ up to 400 nm) upon dissolution of an ASD with 20 wt % DL as soon as the concentration of RIT in the bulk phase exceeds the amorphous solubility (see Figure SI1, Supporting Information). The release profiles thus confirm the complete release of the RIT in RIT/PVPVA ASDs with 20 wt % DL (Figure 5A).

**Table 1:** Amorphous Solubility of RIT, PVPVA, and Vitamin E TPGS in the Aqueous Bulk Phase from Different Experiments at 37 °C

RIT/μg mL^–1^	PVPVA/μg mL^–1^	vitamin E TPGS/μg mL^–1^	ref. to experiment
19.19 (±4.19)	478.03 (±33.62)		Figure 5A
21.69 (±2.95)	457.31 (±76.99)	15.79 (±3.15)	Figure 6A
20.65 (±4.38)	413.38 (±107.62)	17.46 (±1.24)	Figure 6B

At 30 wt % DL (Figure 5B), the release behavior shows a different pattern. Although at a notably slower dissolution rate than identified for a DL of 20 wt %, PVPVA is still completely dissolved. In contrast to the course shown in Figure 5A, the release of RIT is severely negatively impacted. Two findings are obvious: (1) The molecularly dissolved amount of RIT does reach the amorphous solubility only after 90 min in the aqueous bulk phase, and (2) the total released amount of RIT (RIT-rich fragments/droplets and molecularly dissolved RIT) does only reach 38% of the total amount of RIT present in the ASD. Consequently, an incomplete release behavior is observed, whereby after 120 min, 100% of PVPVA but only 38% of the RIT was dissolved/released (LoR).

Even more severe, the releases of both RIT and PVPVA observed after 120 min for a 40 wt % DL (Figure 5C) amount to only 1% of the total amount of RIT in the ASD and 3% of the total amount of PVPVA in the ASD.

### 3.3 Release of ASDs with 3 wt % Vitamin E TPGS

To investigate the influence of Vitamin E TPGS on the presence of RIT-rich fragments and RIT-rich droplets in the aqueous bulk phase, we again investigated the release of ASDs with three different DLs (namely, 20, 30, and 40 wt %) using our improved sampling protocol. For all experiments, 3 wt % Vitamin E TPGS was added to the ASD (replacing an equal amount of polymer). The concentrations of RIT, PVPVA, and Vitamin E TPGS were determined by HPLC (Section 2.4). The hydrodynamic radii (*R*_h_) were measured using DLS (see Section 2.5) to determine the (change in) droplet size and mean droplet diameter in the aqueous bulk phase over time. The results on *R*_h_ are illustrated in Figure SI1 (Supporting Information).

Figure 6A–C illustrates the results on the release behavior of the ASDs containing 3 wt % Vitamin E TPGS at different DLs. For better visualization, only the data points for the total released amounts of Vitamin E TPGS and PVPVA are shown, as released and dissolved amounts were identical. The detailed graphical illustration, including error bars, as well as differentiation between dissolved and release amounts, is shown in Figure SI3 in the Supporting Information.

**Figure 6:**
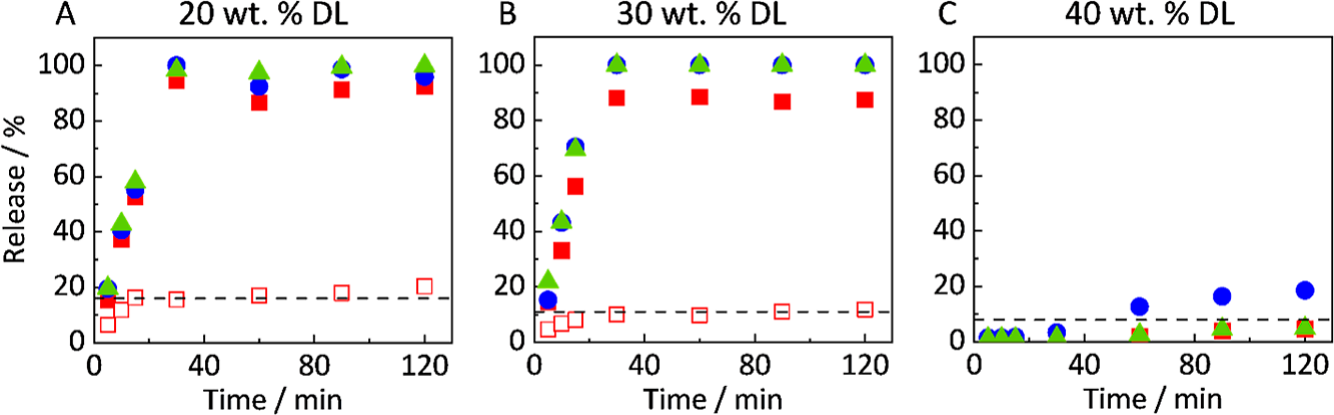
Release profiles of ASDs with 3 wt % Vitamin E TPGS and 20 wt % DL (A), 30 wt % DL (B), 40 wt % DL (C). Filled symbols indicate the released amounts, and empty symbols indicate the molecularly dissolved amounts of RIT (red squares), PVPVA (blue circles), and Vitamin E TPGS (green triangles). The dashed line represents the amorphous solubility of RIT measured in this work.

The results (Figure 6A) reveal that at 20 wt % DL, Vitamin E TPGS and PVPVA were completely dissolved. Similar to the 20 wt % DL ASD without Vitamin E TPGS, RIT is only molecularly dissolved up to its amorphous solubility (see Figure 6A and Table 1). The remaining amount of RIT is present in the released RIT-rich fragments/droplets. As before, particle samples (all time points considered) from the filter contained almost pure RIT, with the concentration of PVPVA and Vitamin E TPGS being below the detection limit of the HPLC (results not shown). Thus, RIT-rich fragments released from the ASD again almost instantaneously change the composition to that of the RIT-rich droplets (in equilibrium with the aqueous bulk phase).

For a 30 wt % DL ASD containing 3 wt % Vitamin E TPGS, the release behavior differs significantly from that of the 30 wt % DL ASD without Vitamin E TPGS. As illustrated in Figure 6B, the addition of Vitamin E TPGS avoids the slowdown of the release (compare Figure 5B). The release behavior is almost identical to that of the 20% DL ASD containing 3 wt % Vitamin E TPGS (Figure 6A). In detail, the RIT-rich droplets in the aqueous bulk phase showed both comparable compositions for the ASDs with 20 wt % DL and 30 wt % DL, as well as comparable *R*_h_ of the droplets (*R*_h_ between 300 and 450 nm, see Figure SI1; Supporting Information). Both 3 wt % Vitamin E TPGS containing ASDs with 20 and 30 wt % DL show rapid and complete RIT release (Figure 6A,B). After 30 min, all components were completely released into the medium.

For 40 wt % DL, the release behavior of the ASD containing 3 wt % Vitamin E TPGS changed significantly and mirrored the behavior of the RIT/PVPVA ASD at 30 wt % DL. The PVPVA dissolution increases after a lag time of 30 min. Only minimal amounts of RIT and Vitamin E TPGS were detected, and both were molecularly dissolved. After 120 min, 20% of PVPVA, 4% of Vitamin E TPGS, and 4% of RIT were released. PXRD experiments revealed that the remaining ASDs were still amorphous after 120 min of the release experiment (Figure SI5, Supporting Information). For all time points considered, the *R*_h_ was measured to <20 nm in the aqueous bulk phase, thus no RIT-rich droplets were formed (Figure SI1, Supporting Information).

### 3.4 Release of ASDs with 40 wt % DL as a Function of the Vitamin E TPGS Content

To determine whether the LoR can be improved for the 40 wt % DL using increasing amounts of Vitamin E TPGS, we investigated two additional ASD compositions containing 7 and 10 wt % Vitamin E TPGS replacing equal amounts of polymer. The release behavior was investigated using our improved sampling protocol. Concentrations of RIT, PVPVA, and Vitamin E TPGS were determined using HPLC, as described in Section 2.4. The *R*_h_ of all measured ASDs can be found in Figure SI1 (Supporting Information). Results are illustrated in Figure 7. For better visualization, only the data points for the total released amounts are shown for Vitamin E TPGS and PVPVA, as released and dissolved amounts were identical. The detailed graphical illustration, including error bars as well as differentiation between dissolved and release amounts, is shown in Figure SI4 in the Supporting Information.

**Figure 7:**
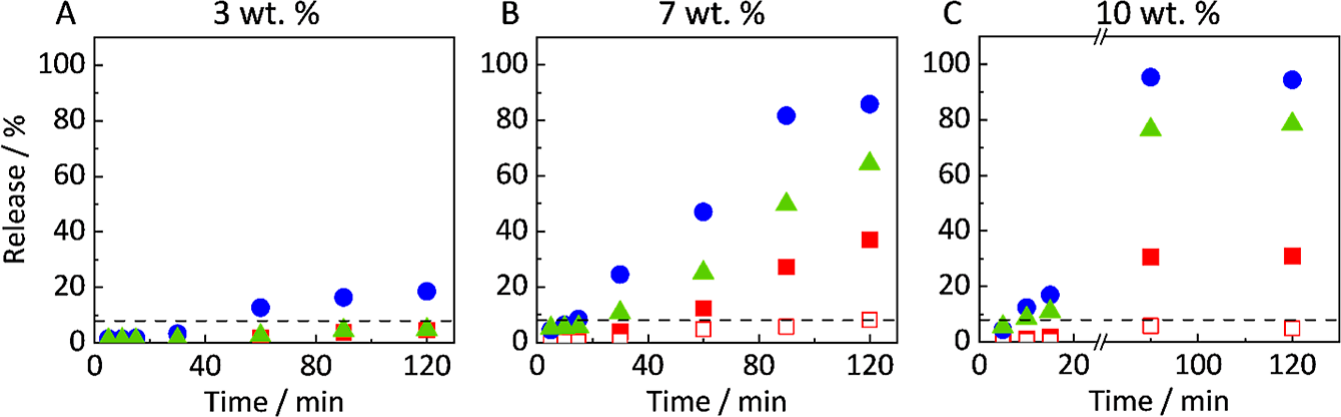
Release profiles of ASDs with 40 wt % DL and 3 wt % (A), 7 wt % (B), or 10 wt % Vitamin E TPGS (C). Filled symbols indicate the released amounts and empty symbols the molecularly dissolved amounts of RIT (red squares), PVPVA (blue circles), and Vitamin E TPGS (green triangles). The dashed line represents the amorphous solubility of RIT measured in this work.

For ASDs with 7 wt % Vitamin E TPGS (Figure 7B), the release behavior is almost identical to that of the 30% DL ASD without Vitamin E TPGS (Figure 5B). An incomplete RIT release is observed with similar concentrations and *R*_h_ (see Figure SI1; Supporting Information). After 120 min, 60–80% of the Vitamin E TPGS and PVPVA and only 35% of the RIT have been released. Therefore, RIT accumulates in the ASD.

Although the release boosts with increasing the Vitamin E TPGS content to 10 wt %, an incomplete RIT release is still observed (Figure 7C). As no lag time is observed in this case, PVPVA and Vitamin E TPGS release faster than from the Vitamin E TPGS-free ASD (30 wt % DL) and from the ASDs with 40 wt % DL and 7 wt % Vitamin E TPGS. During the first 30 min, PVPVA and Vitamin E TPGS releases increase up to 20%, while the release of RIT stagnates at 1%. After an initial lag phase, the release rate of RIT increases until it reaches a plateau after 90 min. After 120 min, only 30% of RIT was released, while 80% of Vitamin E TPGS and the entire PVPVA were released. The remaining ASD thus consisted of about 90 wt % RIT and 10 wt % Vitamin E TPGS. PXRD experiments revealed that the remaining ASDs (starting with 7 and 10 wt % Vitamin E TPGS) were still amorphous after the release experiments (Figure SI5, Supporting Information).

### 3.5 Amorphous Solubility of RIT in the Aqueous Bulk Phase

To identify the impact of the presence of Vitamin E TPGS on the amorphous solubility of RIT, we investigated the maximum (molecularly) dissolved amount of RIT in the aqueous bulk phase. As ASDs with incomplete or collapsed release of RIT did not reach the amorphous solubility of RIT in the aqueous bulk phase, only the solubility data from dissolution experiments with complete release of all components were considered. As described in Section 3.1, the amorphous solubility of RIT was determined after the filtration of all samples. The concentration of RIT was determined via HPLC. The results are shown in Table 1. Accounting for the indicated uncertainty of the measurements, Vitamin E TPGS had no significant impact on the amorphous solubility of RIT in the aqueous bulk phase.

## 4 Discussion

### 4.1 Influence of DL on the Release of Surfactant-Free ASDs

As described in Section 3.2, the release of surfactant-free ASDs decreases with increasing DL. We observed a complete release of RIT at 20 wt % DL, LoR with incomplete RIT release starting at 30 wt % DL, and collapsed release starting at 40 wt % DL (see Figure 5). Thus, the release collapsed upon increasing the hydrophobic-RIT content in the ASD. These findings align with previous studies, which reported an incomplete RIT release of ASDs containing RIT and PVPVA with DLs between 20 and 30 wt % in phosphate buffer (pH = 6.8).^5,6^ Furthermore, our results confirm that a significant amount of the RIT-rich phase remains in the ASD and is not released into the dissolution medium, although PVPVA is completely released after 120 min (see Figure 5B). It is possible that PVPVA breaks through a RIT-rich layer at the ASD-water interface, resulting in complete release, as demonstrated in previous work by Tomberg et al.^15^ using In Situ stimulated Raman microscopy. Increasing DL (>40 wt %) presumably results in a thick RIT-rich layer, preventing PVPVA-rich domains from breaking through^15^ (see Figure 5C).

Particles (fragments and droplets, see Section 3.1) retrieved from the filter were almost free of polymer (see Figure 5), which is in good agreement with the literature findings.^7^ It was thus confirmed that RIT-rich fragments almost instantaneously transform to RIT-rich droplets as soon as the concentration of RIT in the aqueous bulk phase exceeds its amorphous solubility. The RIT-rich droplets present in the media did not exceed sizes of *R*_h_ = 500 nm (see Figure SI1), thus showing no tendency to aggregate/grow in the aqueous bulk phase.

### 4.2 Influence of Vitamin E TPGS on ASD Release

In Figure 6, we showed that adding 3 wt % Vitamin E TPGS to the ASD shifts the start of the LoR from 30 wt % to 40 wt % DL. These observations align with the results of Indulkar et al.,^17^ who could extend the complete RIT release to 28 wt % DL in RIT/PVPVA ASDs by adding 4.7 wt % Vitamin E TPGS. Furthermore, our results reveal that the PVPVA release slightly increases after 30 min in ASDs with 40 wt % DL, suggesting that PVPVA breaks through the RIT-rich layer.^15^

Vitamin E TPGS does not inhibit LLPS in the ASD,^17,18^ which is confirmed by our experiments as an RIT-rich phase remains in the ASD at 40 wt % DL and 7 wt %/10 wt % Vitamin E TPGS (see Figure 7). Previous studies indicated that nonionic surfactants like Tween 80 or Vitamin E TPGS stabilize discrete API-rich domains and hinder the coalescence of the API-rich phase during LLPS in the ASD.^16–18^ Although we did not analyze the compositions of the two phases evolving during LLPS, our findings indicate that Vitamin E TPGS is not enriched in the RIT-rich phase during complete release, as it was determined to be molecularly dissolved in the aqueous bulk phase (see Figures 6 and 7). Nevertheless, Vitamin E TPGS is amphiphilic and can adsorb to the API-polymer interface, caused by LLPS in the ASD.^7^ This leads to the stabilization of discrete RIT-rich domains, which are released simultaneously with the continuous PVPVA-rich phase. However, further studies are required to locate Vitamin E TPGS during LLPS in the ASD, which is part of ongoing work on the topic.

### 4.3 Influence of Vitamin E TPGS on the RIT Solubility in the Aqueous Bulk Phase

It is well-known that surfactants above their critical micelle concentration (CMC) increase the API solubility in an aqueous bulk phase.^24^ In surfactant-free systems, the amorphous solubility of RIT was about 19 μg mL^–1^ (see Table 1). Even at the highest Vitamin E TPGS concentration applied within this work (10 wt % in the ASD), the apparent concentration of Vitamin E TPGS in the aqueous bulk phase did not exceed its CMC (40–50 μg mL^–1^, 37 °C in phosphate buffer pH = 6.8 ^7,20^ ). Therefore, we did not observe an increasing solubility of RIT in the presence of Vitamin E TPGS (see Table 1), which is in good agreement with previous works.^7^

### 4.4 Upper Threshold of Vitamin E TPGS in ASDs

While for a DL of 30 wt %, the presence of 3 wt % Vitamin E TPGS led to complete release of RIT from the ASD (see Figure 6), no complete release could be achieved for ASDs with 40 wt % DL, irrespective of the Vitamin E TPGS concentration applied. Increasing the Vitamin E TPGS content up to 10 wt %, however, led to an unexpected release behavior (see Figure 7). While 100% PVPVA was released and molecularly dissolved, only 80% of the Vitamin E TPGS was released and molecularly dissolved. Additionally, only 20% of RIT is released, with the molecularly dissolved amount reaching a plateau below the amorphous solubility. All remaining ASDs taken from the dissolution apparatus after the release experiments were still amorphous (see PXRD Figure SI5, Supporting Information). These findings suggest a complex phase behavior that may serve as an explanation for the observed failure of Vitamin E TPGS to assist the release mechanism at high DLs.

Figure 8 shows the schematic pseudo ternary phase diagram for the system of RIT/PVPVA at the fixed ratio of RIT to PVPVA 40:60 (ASD40), Vitamin E TPGS, and water. For the first time, this diagram provides insight into the phase separation of the quaternary system based on the phase separation observed in binary systems. It is known that ASD40 and water exhibit an LLPS at 37 °C^21^ (see Figure 8). For the binary system composed of ASD40 and Vitamin E TPGS, no experimental data on LLPS are available. However, recent investigations suggest phase separation in binary mixtures of PVPVA and Vitamin E TPGS at Vitamin E TPGS contents higher than 7 wt %.^25,26^ As ASD40 is rich in PVPVA, we thus also assume an LLPS in the Vitamin E TPGS/ASD40 system (see Figure 8). The binary system of Vitamin E TPGS and water does not show an LLPS at 37 °C. However, LLPS starts occurring at 78 °C.^27^ Taking these considerations for the three binary subsystems into account, it is plausible that the concentration regions of binary LLPS overlap/intersect in the quaternary system, thus creating a huge concentration range where three liquid phases exist in equilibrium (LLLPS) (Figure 8), each of which is rich in either water, Vitamin E TPGS, or RIT/PVPVA.

**Figure 8:**
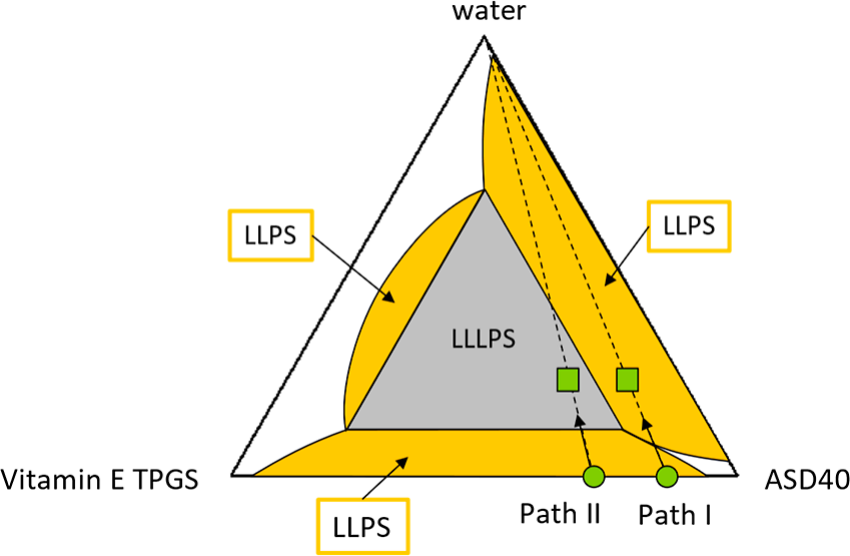
Schematic phase diagram of ASD40, TPGS, and water. White areas are homogeneous mixtures, yellow areas represent phase separation into two liquid phases (LLPS), and the gray area represents phase separation into three liquid phases (LLLPS). Path I indicates the hydration pathway of an ASD with a Vitamin E TPGS content below the threshold, and path II the hydration pathway of an ASD with a Vitamin E TPGS content above a threshold. The green circles indicate a dry ASD at the beginning of the release experiments, and the green squares indicate the demixing point after water sorption during release experiments.

Two pathways (Path I and Path II) can now be differentiated when the dry ASD is brought into contact with the aqueous bulk phase. If the Vitamin E TPGS content in the ASD is below the upper threshold, the phase separation behavior will adhere to Path I in Figure 8. This will lead to the usually expected LLPS (only two phases) behavior and, depending on which phase is the continuous phase (see Figures 2 and 3), either the desired release behavior (Figure 6, ASD with 3 wt % Vitamin E TPGS and 30 wt % DL) or a LoR (Figure 7, ASD with 3 wt % Vitamin E TPGS and 40 wt % DL).

If the upper Vitamin E TPGS threshold is exceeded (e.g., 10 wt % Vitamin E TPGS, see Figure 7), the separation profile adheres to Path II in Figure 8. In this case, the ASD separates into three amorphous phases: a RIT-rich phase, a PVPVA-rich phase, and a Vitamin E TPGS-rich phase (path II in Figure 8). As a result, the previous mechanism fails, as the coalescence of the RIT-rich phase can no longer be inhibited by Vitamin E TPGS (due to the formation of a Vitamin E TPGS-rich phase). This also could explain the observation that, although PVPVA is released at 100%, only 80% of Vitamin E TPGS is released (see Figure 7C) for the 40 wt % DL ASD containing 10 wt % Vitamin E TPGS. Thus, 20% of the Vitamin E TPGS remains in the ASD as Vitamin E TPGS-rich phase. The phase diagram also indicates that further increasing the TPGS content beyond this threshold (e.g., 10 wt % Vitamin E TPGS) does not change the phase composition until nearly the entire formulation consists of Vitamin E TPGS.

### 4.5 Mechanism of Vitamin E TPGS-Assisted Release

Based on our release experiments and the discussions above, our hypothesis on the mechanism of Vitamin E TPGS-assisted release is illustrated in Figure 9. We distinguish three cases that may happen when adding Vitamin E TPGS to an RIT/PVPVA ASD with a DL above the LoR.

**Figure 9:**
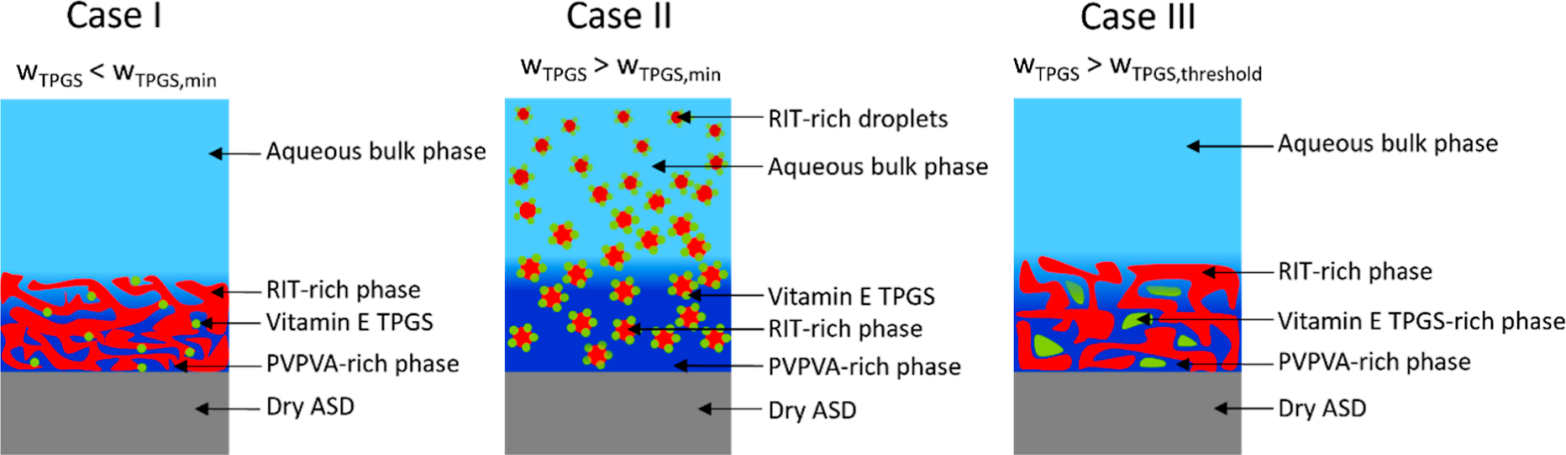
Schematic illustration of the Vitamin E TPGS-assisted release mechanism of RIT/PVPVA ASDs. Case I: The ASD contains an insufficient amount of Vitamin E TPGS. Case II: The ASD contains a sufficient amount of Vitamin E TPGS. Case III: The ASD contains an amount of Vitamin E TPGS that exceeds the upper threshold.

In Case I, the addition of an insufficient amount of Vitamin E TPGS cannot prevent incomplete or even collapsed RIT release. Although Vitamin E TPGS can adsorb at the RIT-PVPVA interface during LLPS in the ASD, the amount of Vitamin E TPGS molecules is insufficient to stabilize discrete RIT-rich domains. Thus, the initial RIT-rich domains coalesce almost instantaneously during LLPS in the ASD, forming a continuous RIT-rich phase and a discrete PVPVA-rich phase. Like for surfactant-free ASDs (see ′LoR′ in Section 1), PVPVA dissolves into the aqueous bulk phase, while the RIT-rich phase remains as a porous layer of the ASD. This hinders further dissolution of all components, resulting in LoR (see Figure 7A, ASD with 3 wt % Vitamin E TPGS and 40 wt % DL).

In Case II, the increased amount of Vitamin E TPGS molecules now enables the stabilization of discrete RIT-rich domains during LLPS in the ASD, hindering their coalescence. The PVPVA-rich phase dissolves completely, and the RIT-rich phase dissolves until the amorphous solubility of RIT in the aqueous bulk phase is reached (see Figure 6B, ASD with 3 wt % Vitamin E TPGS and 30 wt % DL). After that, the RIT-rich phase is released into the aqueous bulk phase in the form of RIT-rich fragments (see “Desired release behavior” in surfactant-free ASDs, Section 1). These fragments then quickly transform into RIT-rich droplets in the aqueous bulk phase (see Section 3.3 and Figure SI1, Supporting Information). Thus, the addition of sufficient Vitamin E TPGS leads to the desired complete RIT release over time.

In Case III, the amount of Vitamin E TPGS in the ASD exceeds the upper threshold (Section 4.4). Instead of being present in one/or both of the two LLPS phases and thus being able to adsorb at the RIT–PVPVA interface, Vitamin E TPGS now forms a distinct third phase rich in Vitamin E TPGS. This phase does not participate in the aforementioned stabilization mechanism. Due to the presence of the third phase, the amount of Vitamin E TPGS in the RIT-rich and the PVPVA-rich phases is reduced significantly (to values that now follow Case I). This leads to an incomplete RIT release (see Figure 7C, ASD with 10 wt % Vitamin E TPGS and 40 wt % DL). However, as the continuous network of the RIT-rich phase is now more porous (both the PVPVA-rich phase and Vitamin E TPGS-rich phase dissolve faster), more RIT is released compared to the LoR observed in the Vitamin E TPGS-free system (Figure 5). We assume that this release behavior could also be observed in biological systems with higher shear rates, as increased rotation speed might accelerate the release^28^ but would not affect the thermodynamic equilibrium.

Summing up, the release experiments reveal three distinct cases for Vitamin E TPGS-assisted release in RIT/PVPVA ASDs. In a certain concentration range, Vitamin E TPGS (Case II) stabilizes RIT-rich domains, enabling their controlled dissolution and leading to the desired release. In contrast, an insufficient amount of Vitamin E TPGS (Case I) fails to stabilize discrete RIT-rich domains, leading to their coalescence and formation of a continuous RIT-rich phase, which hinders complete release. However, adding excessive amounts of Vitamin E TPGS induced the formation of the third phase (Case III), reducing its stabilizing potential within the other phases and resulting in incomplete RIT release. The schematic phase diagram suggests that even if further increasing the TPGS content, the phase composition remains unchanged until nearly the entire formulation consists of Vitamin E TPGS. Therefore, “the more Vitamin E TPGS, the better” does not apply.

We suggest that these results can be extrapolated to release experiments conducted in intestinal fluids at pH 6.8, as our results are consistent with previous investigations performed in phosphate buffer (pH = 6.8).^17^ The presence of additional substances such as salts in biological fluids can affect phase separation in the ASD, which was not considered in this work. We furthermore hypothesize that the proposed release mechanisms may also be applicable to ASDs formulated with other APIs (e.g., the acid API indomethacin) as well as alternative surfactants, provided that the binary systems of API/water, polymer/surfactant, and water/surfactant exhibit phase separation, and the API does not undergo rapid recrystallization in the ASD.

## 5 Conclusion

In this work, we developed an easy-to-use sampling protocol to gain detailed insight into the release behavior of ASDs to distinguish between the released and molecularly dissolved amounts of the ASD components. We were able to confirm the previous investigation on the Vitamin E TPGS-assisted release behavior for RIT/PVPVA ASDs with DLs up to 30 and 3 wt % Vitamin E TPGS. Our results suggest that Vitamin E TPGS acts as a surface-active agent at the RIT-PVPVA interface during LLPS. It stabilizes distinct RIT-rich phase domains, hindering their coalescence and enabling their simultaneous release with the continuous PVPVA-rich phase.

However, our results also reveal the limitations of this stabilization mechanism, as at elevated concentrations of Vitamin E TPGS (about 10 wt %), congruent release of all three components could not be achieved for ASDs with DLs higher than 40 wt %. This suggests that beyond an upper threshold, the surfactant is no longer able to stabilize RIT-rich domains. As a probable reason for that, we hypothesize the formation of a third phase rich in Vitamin E TPGS during phase separation in the ASD. This phase behavior disrupts the stabilization mechanism that was effective until then, leading again to incongruent and incomplete release of RIT. To the best of our knowledge, this is the first report of a three-phase demixing in ASDs. Further insights into the three-phase demixing using Raman spectroscopy will be presented in a future publication.

This study thus underscores the critical role of surfactants like Vitamin E TPGS in optimizing ASD formulations while also highlighting the challenges that arise at high surfactant concentrations. We showed that for Vitamin E TPGS in Ritonavir/PVPVA ASDs, “the more the better” does not apply and provided a mechanistic understanding of surfactant-assisted release behavior. These insights are valuable for the development of formulations in which optimizing API release must be balanced with other critical factors, such as long-term stability or manufacturability.

## Supplementary Material



## Data Availability

Data are contained within the Article or Supporting Information.

## Notes

Hydrodynamic radius (*r*_h_) of API-rich droplets, release profiles of all ASDs, PXRD pattern of the remaining ASDs after the release experiments, mDSC Analysis of an ASDs with 40 wt % DL and 10 wt % Vitamin E TPGS, and validation of the ASD sampling protocol (PDF)
