# Supplementary material for: The More the Better?—Vitamin E TPGS as a Release Enhancer for Ritonavir/PVPVA Amorphous Solid Dispersions
Source: Mol Pharm. 2025 Jul 27;22(9):5592–602. doi: 10.1021/acs.molpharmaceut.5c00620 (PMC12406244; doi:10.1021/acs.molpharmaceut.5c00620)
Supplement: Supplementary file 1 [file mp5c00620_si_001.pdf]

The more the better? – Vitamin E TPGS as release enhancer for Ritonavir/PVPVA ASDs

Ineke Fahrig[1], *Stefanie Walter*[2], *Samuel Kyeremateng*[2*], *Matthias Degenhardt*[2], *Gabriele Sadowski*[1*], *Christoph Brandenbusch*[1]

[1] TU Dortmund University, Department of Biochemical and Chemical Engineering, Laboratory of Thermodynamics, Emil-Figge-Str. 70, D-44227 Dortmund, Germany

[2] AbbVie Deutschland GmbH & Co. KG, Development Sciences, R&D, Knollstraße, D-67061 Ludwigshafen am Rhein, Germany

[*]*corresponding authors: gabriele.sadowski@tu-dortmund.de*

samuel.kyeremateng@abbvie.com

Supporting information

1.1 Hydrodynamic radius of API-rich droplets

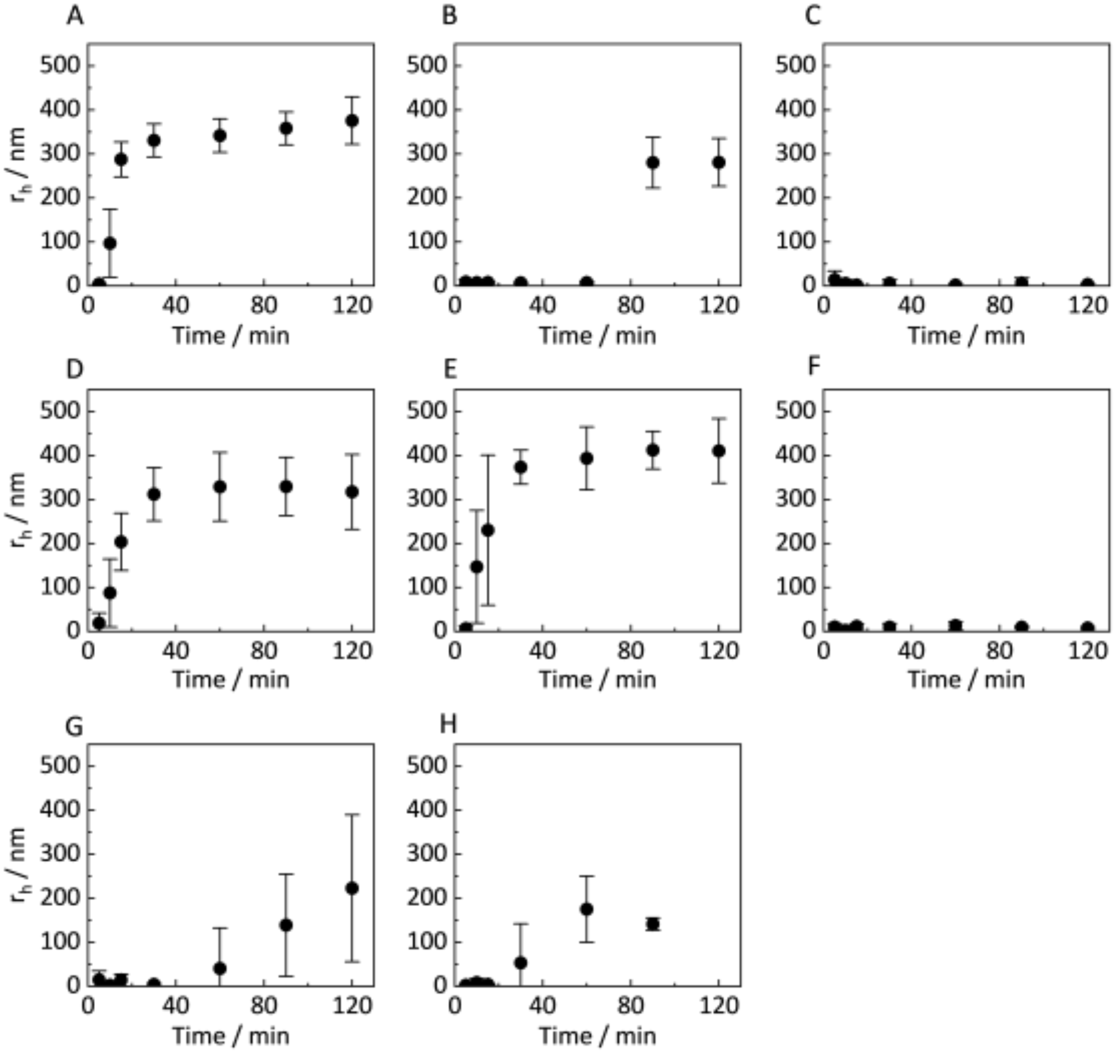

Figure SI-1. Hydrodynamic radius (rh) of API-rich droplets in ASDs with 20 wt. % DL and 0 wt. % Vitamin E TPGS (A), 30 wt. % DL and 0 wt. % Vitamin E TPGS (B), 40 wt. % DL and 0 wt. % Vitamin E TPGS (C), 20 wt. % and 3 wt. % Vitamin E TPGS (D), 30 wt. % DL and 3 wt. % Vitamin E TPGS (E), 40 wt. % DL and 3 wt. % Vitamin E TPGS (F), 40 wt. % DL and 7 wt. % Vitamin E TPGS (G), 40 wt. % DL and 10 wt. % Vitamin E TPGS (H).

1.2 Release profiles

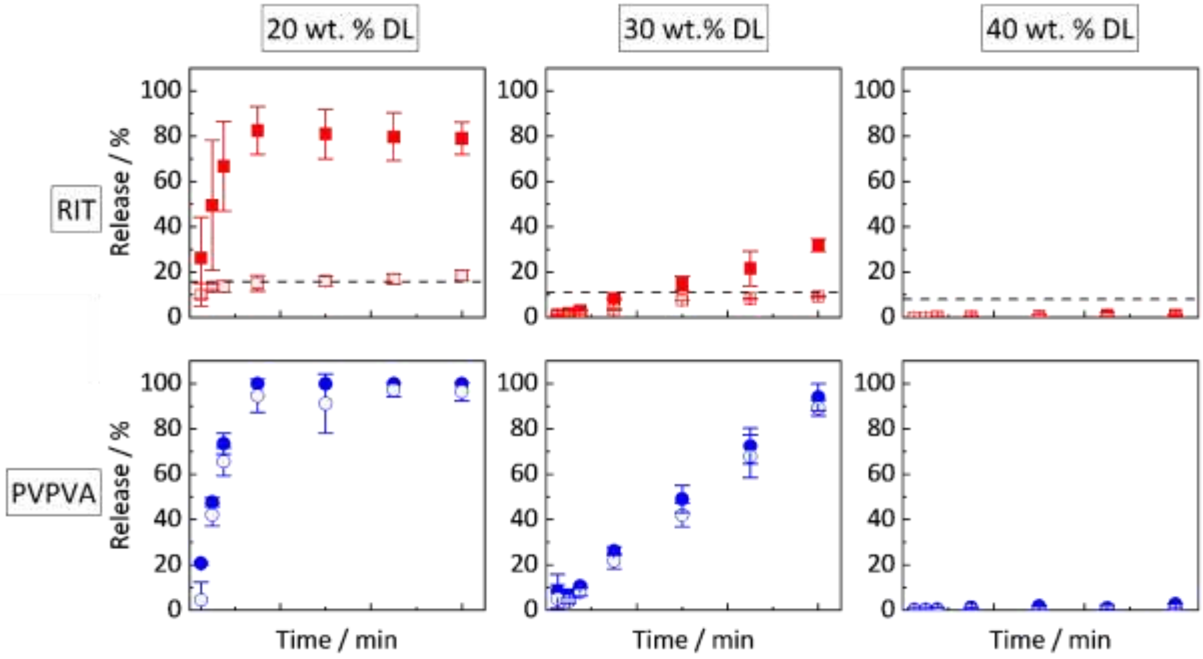

Figure SI- 2. Release profiles of RIT/PVPVA ASDs with variable DL. Filled symbols indicate the release amount and empty symbols the molecularly dissolved amount of all components. The dashed line represents the amorphous solubility determined in this work.

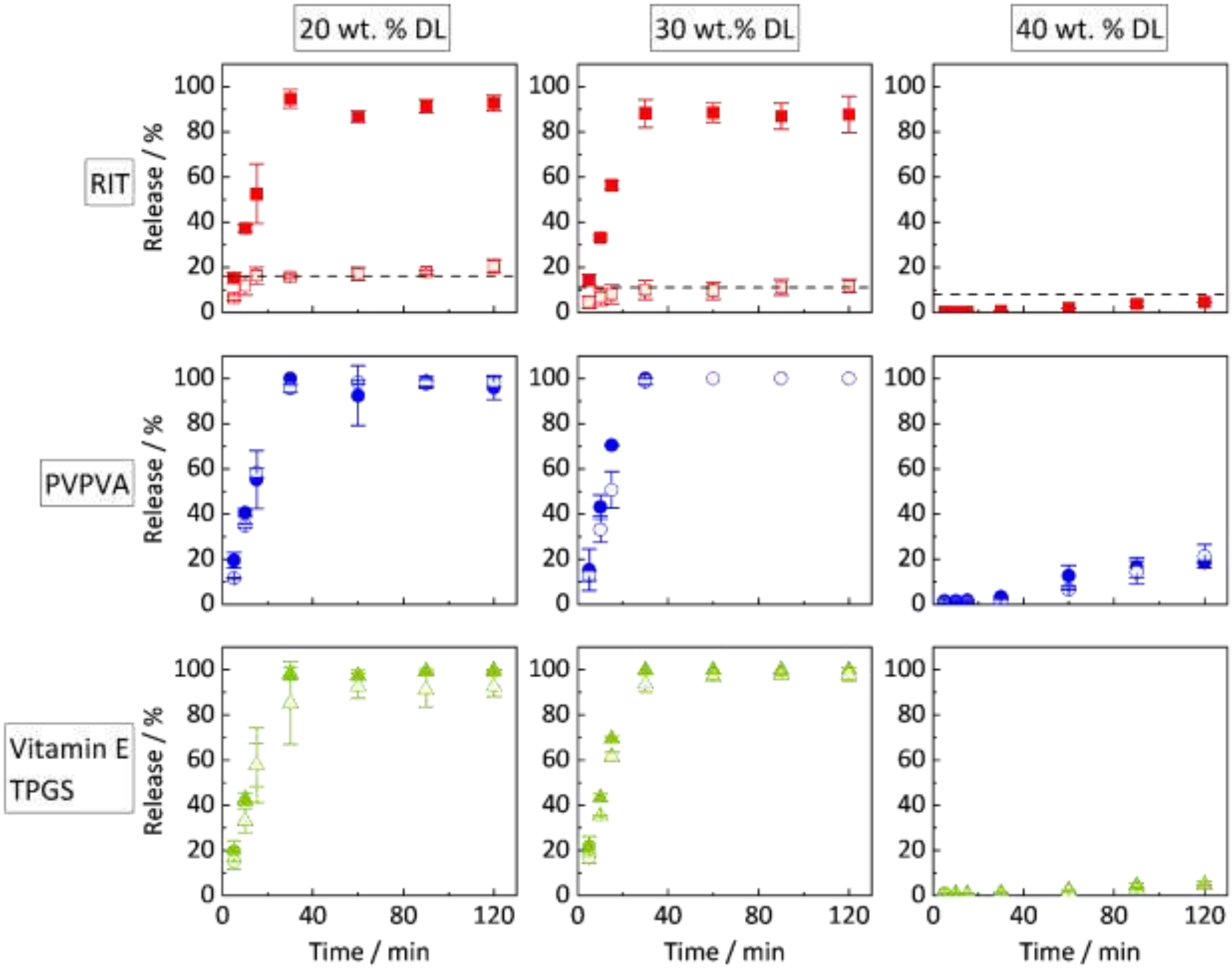

Figure SI- 3. Release profiles of ASDs with 3 wt. % Vitamin E TPGS and variable DL. Filled symbols indicate the release amount and empty symbols the molecularly dissolved amount of all components. The dashed line represents the amorphous solubility of RIT determined in this work.

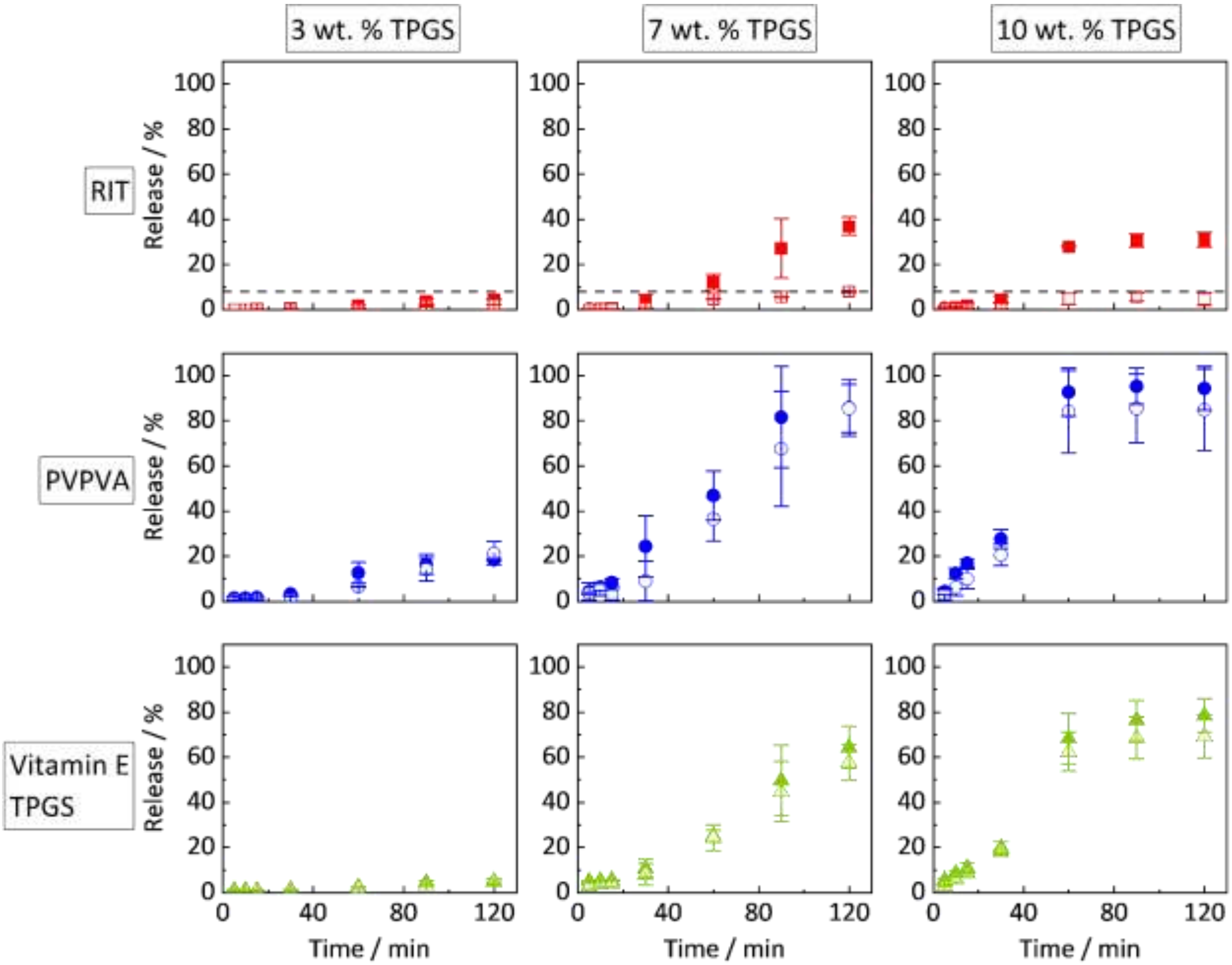

Figure SI- 4. Release profiles of ASDs with 40 wt. % DL and variable Vitamin E TPGS content. Filled symbols indicate the release amount and empty symbols the molecularly dissolved amount of all components. The dashed line represents the amorphous solubility of RIT determined in this work.

1.3 PXRD pattern of remained ASDs

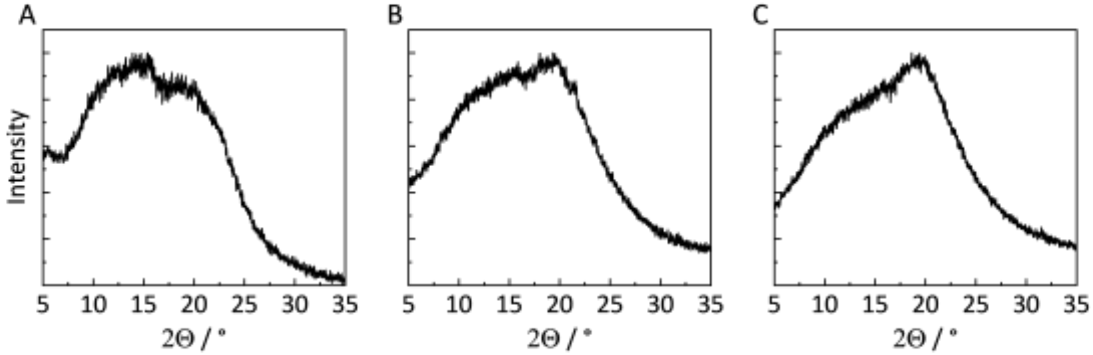

Figure SI-5. PXRD pattern of remained ASDs after the release experiments. A: 40 wt. % DL, 0 wt. % Vitamin E TPGS, B: 40 wt. % DL, 7 wt. % Vitamin E TPGS, C: 40 wt. % DL and 10 wt. % Vitamin E TPGS

1.4 mDSC Analysis

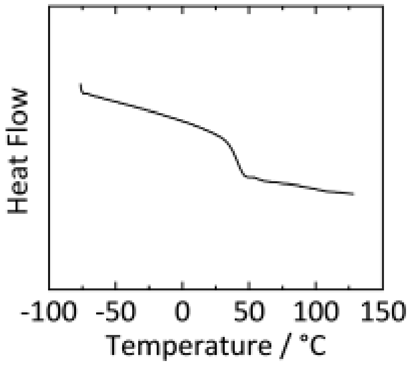

Figure SI- 6. mDSC analysis of an ASD with 40 wt. % DL and 10 wt. % Vitamin E TPGS after preparation. The measurement was conducted using a DSC2000 instrument from TA Instruments-Waters LLC (Newcastle, USA) with a heating rate of 5 K min^{-1}.

1.5 Validation of the ASD sampling protocol

To ensure that the improved sampling method provides representive results for the dissolved amounts of all components, we assessed both potential adsorption to the filter and the mass balance after filtration.

Since PVPVA and Vitamin E TPGS are completely soluble in the aqueous bulk phase, we focused on the adsorption behavior of RIT. To investigated the potential adsorption to the filter membrane, a standard solution of RIT (0.15 g L^{-1} in methanol) was prepared. Aliquots (1 mL) of this solution were filtered through a 0.1 µm PVDF syringe filter (Berrytech GmbH, Harthausen, Germany) into clean Eppendorf tubes. Both filtered and unfiltered samples were analyzed using HPLC, as described in Section 2.4 óf the main manuscript. The recovery, calculated as the ratio of RIT concentration in the filtered samples to that in the unfiltered samples, was found to be 99%, indicating negligible adsorption to the filter.

We furthermore quantified the concentration and mass of particles retained on the filter to assess mass balance. This validation was conducted using ASDs containing 20 wt. % DL and 0 wt. % Vitamin E TPGS. During release experiments, 5 mL samples were withdrawn at defined time points and divided into two aliquots, as explained in section 3.1 of the main manuscript. The first aliquot (1 mL) was not filtered but diluted 1:1 v/v % with methanol to determine the total amount of each component in the aqueous bulk phase. The second aliquot (2 mL) was filtered through the 0.1 µm PVDF syringe filter. The filtrate was analyzed by HPLC to quantify the dissolved fractions of the components without further dilution.

Particles retained on the filter were recoverd by dissolving with methanol and analyzed by HPLC. The concentration of PVPVA was below the detection limit in the HPLC, confirming

the complete dissolution in the aqueous phase, consistent with the release profiles. In contrast, a distinct RIT peak was detected, indicating that the retained particles primarily consisted of undissolved RIT. Furthermore, the mass of recovered RIT from the filter matched the mass difference between the total and dissolved RIT amounts, confirming mass balance and validating the accuracy of the filtration-based sampling protocol (see Table SI-1).

Table SI-1. Mass of the filtered particles during release of an ASD with 20 wt. % DL and 0 wt. % Vitamin E TPGS. m_{total} -$m_{dissolved}$ describe mass difference between the total (release) and dissolved amount and $m_{recovered}$ the mass of the retained particles in the filter.

**Time / min**	**(m_{total} -$m_{dissolved}$) / mg**	**$m_{recovered}$ / mg**
5	0.7	0.5
10	2.4	1.5
15	4.9	3.6
30	6.0	6.3
60	5.9	5.5
90	5.6	5.2
120	6.4	5.2
